# Diversity and phylogeny of the tick-borne bacterial genus *Candidatus* Allocryptoplasma (Anaplasmataceae)[Fn FN1]

**DOI:** 10.1051/parasite/2023014

**Published:** 2023-05-10

**Authors:** Sofian Ouass, Nathalie Boulanger, Benjamin Lelouvier, Jean-Louis-Marie Insonere, Camille Lacroux, Sabrina Krief, Edward Asalu, Nil Rahola, Olivier Duron

**Affiliations:** 1 MIVEGEC, University of Montpellier (UM), Centre National de la Recherche Scientifique (CNRS), Institut pour la Recherche de la Développement (IRD) 34394 Montpellier France; 2 University of Strasbourg, French National Reference Center for Borrelia, Hôpitaux Universitaires de Strasbourg Strasbourg France; 3 Vaiomer 516 rue Pierre et Marie Curie 31670 Labège France; 4 UMR 7206 CNRS/MNHN/P7, Eco-anthropologie, Muséum National d'Histoire Naturelle, Musée de l'Homme 17 place du Trocadéro 75116 Paris France; 5 La Phocéenne de Cosmétique, ZA Les Roquassiers 174 Rue de la Forge 13300 Salon-de-Provence France; 6 Sebitoli Chimpanzee Project, Great Ape Conservation Project Kibale National Park Fort Portal Uganda; 7 Uganda Wildlife Authority, Kibale National Park Uganda

**Keywords:** Anaplasmataceae, Anaplasma, Ehrlichia, Allocryptoplasma, Tick, Tick-borne diseases

## Abstract

The family Anaplasmataceae includes tick-borne bacteria of major public and veterinary health interest, as best illustrated by members of the genera *Anaplasma* and *Ehrlichia*. Recent epidemiological surveys have also reported on the presence of a novel putative genus in the Anaplasmataceae, *Candidatus* Allocryptoplasma, previously described as *Candidatus* Cryptoplasma in the western black-legged tick, *Ixodes pacificus*. However, the genetic diversity of *Ca.* Allocryptoplasma and its phylogenetic relationship with other Anaplasmataceae remain unclear. In this study, we developed a multi-locus sequence typing approach, examining the DNA sequence variation at five genes of *Ca.* Allocryptoplasma found in ticks. Combining this multi-locus sequence typing and genetic data available on public databases, we found that substantial genetic diversity of *Ca.* Allocryptoplasma is present in *Ixodes*, *Amblyomma* and *Haemaphysalis* spp. ticks on most continents. Further analyses confirmed that the *Ca.* Allocryptoplasma of ticks, the *Ca.* Allocryptoplasma of lizards and some *Anaplasma*-like bacteria of wild mice cluster into a monophyletic genus, divergent from all other genera of the family Anaplasmataceae. *Candidatus* Allocryptoplasma appears as a sister genus of *Anaplasma* and, with the genera *Ehrlichia* and *Neoehrlichia*, they form a monophyletic subgroup of Anaplasmataceae associated with tick-borne diseases. The detection of genetically distinct *Ca.* Allocryptoplasma in ticks of significant medical or veterinary interest supports the hypothesis that it is an emergent genus of tick-borne pathogens of general concern.

## Abstract (fr)

La famille des Anaplasmataceae comprend des bactéries transmises par les tiques qui présentent un intérêt majeur pour la santé publique et vétérinaire, comme les membres des genres *Anaplasma* et *Ehrlichia*. Des surveillances épidémiologiques récentes ont également signalé la présence d'un nouveau genre putatif dans les Anaplasmataceae, *Candidatus* Allocryptoplasma, initialement décrit comme *Ca.* Cryptoplasma chez une tique nord-américaine, *Ixodes pacificus*. Cependant, la diversité génétique des bactéries *Ca.* Allocryptoplasma et leurs relations phylogénétiques avec d'autres Anaplasmataceae restent méconnues. Dans cette étude, nous avons développé une approche de typage génétique multi-locus, en examinant la variation nucléotidique pour cinq gènes de bactéries *Ca.* Allocryptoplasma détectées chez les tiques. En combinant ce typage génétique multi-locus et les données génétiques disponibles dans les bases de données publiques, nous avons mis en évidence qu’une diversité génétique substantielle des bactéries *Ca.* Allocryptoplasma est présente chez les tiques des genres *Ixodes*, *Amblyomma* et *Haemaphysalis* sur la plupart des continents. Des analyses complémentaires confirment que les bactéries *Ca.* Allocryptoplasma des tiques, les bactéries *Ca.* Allocryptoplasma de lézards et des bactéries *Anaplasma*-like de souris sauvages se regroupent dans un genre monophylétique, divergent de tous les autres genres de la famille Anaplasmataceae. *Candidatus* Allocryptoplasma apparaît comme un genre frère d'*Anaplasma* et, avec les genres *Ehrlichia* et *Neoehrlichia*, ces trois genres forment un sous-groupe monophylétique d'Anaplasmataceae associé aux maladies transmises par les tiques. La détection de *Ca.* Allocryptoplasma dans des tiques d'intérêt médical et vétérinaire soutient l'hypothèse qu'il s'agit d'un genre émergent d'agents pathogènes majeurs.

## Introduction

Members of the family Anaplasmataceae (Order: Rickettsiales) are obligate intracellular bacteria that can infect a wide range of animals [10, 36]. Historically, two genera, *Anaplasma* and *Ehrlichia*, have been described as important tick-borne bacteria of public and veterinary health interest [1, 3, 11, 12, 18, 33]. These genera include *Anaplasma phagocytophilum* and *Ehrlichia chaffeensis* that cause human granulocytic anaplasmosis and human monocytic ehrlichiosis, respectively and *Ehrlichia ruminantium* that causes heartwater (cowdriosis) in African cattle [1, 11, 12, 18, 33]. Recent advances in the genomics of these major pathogens have shed new light on the intracellular lifestyle of Anaplasmataceae and the mechanisms by which they induce and evade the innate immune response [2, 15, 17, 25]. Current epidemiological surveys on Anaplasmataceae are now uncovering emergent tick-borne pathogens such as *Neoehrlichia mikurensis*, causing neoehrlichiosis in Europe and Asia [31, 38], and *Ca.* Anaplasma sparouinense, causing Sparouine anaplasmosis in South America [13]. Other pathogens in the family Anaplasmataceae include *Aegyptianella pullorum*, which has only been observed once in domestic turkeys [35], and *Neorickettsia* spp. of digeneans (Platyhelminthes), which can be transmitted to the vertebrate hosts of digeneans causing Sennetsu fever in humans (*Neorickettsia sennetsu*) and Potomac fever in horses (*Neorickettsia risticii*) [41, 42]. However, the family Anaplasmataceae also includes bacteria that cannot infect vertebrates: *Ca.* Xenohaliotis californiensis and *Ca.* Xenolissoclinum pacificiensis, both described from marine invertebrates [16, 21], and members of the *Wolbachia* genus, specific to terrestrial arthropods and filarial nematodes [19, 23].

An intriguing and little known member of the Anaplasmataceae family is the putative genus *Ca.* Allocryptoplasma. It was created to name a bacterium originally described as *Ca.* Cryptoplasma californiense [14], but further corrected to *Ca.* Allocryptoplasma californiense (the generic name *Cryptoplasma* was already in use in the nomenclature of protozoa) [30]. In 2015, *Candidatus* Allocryptoplasma californiense was described in western black-legged ticks, *Ixodes pacificus*, collected throughout California [14]. *Candidatus* Allocryptoplasma californiense was detected in 5% of *I. pacificus* ticks tested statewide, but its prevalence was as high as 21% at one site [14]. Early sequence analysis indicated that *Ca.* Allocryptoplasma californiense is phylogenetically distinct from known genera and species from the Anaplasmataceae family, although genetically related to an *Anaplasma*-like bacterium detected in Asian longhorned ticks, *Haemaphysalis longicornis*, in Korea and China [14, 29, 32]. Further examination of partial 16S rRNA gene sequences showed high levels of homology to *Ca.* Allocryptoplasma californiense for Anaplasmataceae bacteria detected in castor bean ticks, *Ixodes ricinus*, in France [34], Italy [27], Slovakia [20], Serbia [4], Tunisia and Morocco [37]. Diverse strains of *Ca.* Allocryptoplasma were also detected in *Haemaphysalis parmata* and *Amblyomma tholloni* ticks collected in a wild chimpanzee habitat in Uganda [22], *Amblyomma dissimile* in Brazil [28] and in harvest mites of lizards, *Neotrombicula autumnalis*, in Italy [27]. While these bacteria have never been isolated, DNA of similar *Ca.* Allocryptoplasma sp. and *Anaplasma*-like bacteria were also detected in vertebrates which could act as primary hosts for their maintenance and enzootic circulation in Europe. These bacteria were detected in striped field mice, *Apodemus agrarius* [40], and green lizards, *Lacerta viridis* [20], in Slovakia, and in common wall lizards, *Podarcis muralis*, Italian wall lizards, *Podarcis siculus*, and western green lizards, *Lacerta bilineata*, in Italy [27]. Although the genus *Ca.* Allocryptoplasma has not been validly published to date, its detection in tick species of public and veterinary health interest, mammals and reptiles suggests that it could be a major genus of tick-borne pathogens.

Despite the potential medical and veterinary importance of the genus *Ca.* Allocryptoplasma, the phylogenetic relationship and pattern of genetic variation between *Ca.* Allocryptoplasma californiense, potential *Ca.* Allocryptoplasma spp., closely related *Anaplasma*-like bacteria and other members of Anaplasmataceae family remain unclear. Here, we address these issues by characterizing genetic variation of *Ca.* Allocryptoplasma infections and by conducting phylogenetic inferences to reconstruct their evolutionary histories within the family Anaplasmataceae. While 16S rRNA, *groEL*, *rpoB* and *gltA* gene sequences were used to describe *Ca.* Allocryptoplasma californiense in *I. pacificus* ticks [14], only partial 16S rRNA gene sequences have been used as exclusive markers for description of other *Ca.* Allocryptoplasma sp. and *Anaplasma*-like bacteria [4, 20, 22, 27–29, 32, 37, 40]. However, the use of 16S rRNA gene sequences as an exclusive taxonomic marker was recently shown to be inadequate for inferring a reliable phylogeny within the Anaplasmataceae since the tree topology is often poorly resolved and usually unstable because of insufficient sequence polymorphism [7]. We therefore developed, in the present study, a generic multi-locus sequence typing approach, examining the DNA sequence variation of *Ca.* Allocryptoplasma spp. at five genes (16S rRNA, *groEL*, *rpoB*, *gltA* and *sucA*) and applied this typing to the strains we detected in four tick species sampled in Europe, South America, and Africa. Using this multi-locus typing approach and data available on public databases, we further examined the phylogenetic placement of *Ca.* Allocryptoplasma californiense and other *Ca.* Allocryptoplasma spp. within the family Anaplasmataceae.

## Materials and methods

### Ethics

The use of ticks from French Guiana was approved by the French Ministry of the Environment under the reference #TREL19028117S/156, in compliance with the Access and Benefit Sharing procedure implemented by the *Loi pour la Reconquête de la Biodiversité*. The research in Uganda was conducted in the context of the Memorandum of Understanding “Museum National d'Histoire Naturelle/Uganda Wildlife Authority/Makerere University SJ 445-12”, in accordance with the Uganda Wildlife Authority and the Uganda National Council for Science and Technology.

### Tick collection

A collection of 26 individual DNA templates obtained from 26 specimens of four tick species was used (Table S1). DNA was extracted from individual ticks using a DNeasy Blood & Tissue Kit (QIAGEN), following the manufacturer’s instructions. For each DNA template, tick identification and infection by *Ca.* Allocryptoplasma sp. have been formally characterized in previous investigations by molecular and morphological characteristics (for ticks) and high-throughput 16S rDNA sequencing approach for microbiota profiling (for *Ca.* Allocryptoplasma sp.) (Table S1). The four infected tick species examined in this study belong to the Ixodidae family (hard ticks), and include *Amblyomma coelebs* (one specimen), *A. tholloni* (12 specimens), *H. parmata* (three specimens) and *I. ricinus* (10 specimens) (Table S1). All tick specimens were questing (non-engorged) nymphs or adults collected from vegetation in South America (*A. coelebs*), Africa (*A. tholloni*, *H. parmata*), and Europe (*I. ricinus*) (Table S1).

### Multi-locus typing of *Ca.* Allocryptoplasma infection

*Ca.* Allocryptoplasma sp. were genotyped through nested or semi-nested PCR assays and by sequencing of five housekeeping genes (16S rRNA [1187 bp], *rpoB* [496 bp], *sucA* [548–616 bp], *groEL* [572 bp], and *gtlA* [606-977 bp]). For 16S rRNA, we used our previously published primers (listed in Table S2), which were previously designed for *Ehrlichia* typing but were further found to be effective in amplifying *Ca.* Allocryptoplasma sp. [22]. For the other four genes, *Ca.* Allocryptoplasma-specific primers (Table S2) were designed using *Anaplasma* and *Ehrlichia* reference genomes available in public databases (GenBank accession numbers: *A. phagocytophilum*, CP006617; *A. marginale*, CP001079; *A. centrale*, CP001759; *E. chaffeensis*, CP007480; *E. ruminantium*, CP040111; *E. canis*, CP000107), and *groEL*, *rpoB* and *gltA* gene sequences (GenBank accession numbers: KP276592–KP276592, KP276600–KP276602, KP276604–KP276606) primarily used to describe *Ca.* Allocryptoplasma californiense in *I. pacificus* ticks [14]. These genes are found as single copies in *Anaplasma* and *Ehrlichia* reference genomes.

Nested and semi-nested PCR amplifications were performed as follows: the first PCR run with the external primers was performed in a 10 μL volume containing 10–50 ng of genomic DNA, 3 mM of each dNTP (Thermo Scientific), 8 mM of MgCl_2_ (Roche Diagnostics), 3 μM of each primer, 1 μL of 10× PCR buffer (Roche Diagnostics), and 0.5 U of Taq DNA polymerase (Roche Diagnostics). A 1 μL aliquot of the PCR product from the first reaction was then used as a template for the second round of amplification. The second PCR was performed in a total volume of 25 μL and contained 8 mM of each dNTP (Thermo Scientific), 10 mM of MgCl_2_ (Thermo Scientific), 7.5 μM of each of the internal primers, 2.5 μL of 10×PCR buffer (Thermo Scientific), and 1.25 U of Taq DNA polymerase (Thermo Scientific). All PCR amplifications were performed as follows: initial denaturation at 93 °C for 3 min, 35 cycles of denaturation (93 °C, 30 s), annealing (*T*_m_ = 52 °C), extension (72 °C, 1 min), and a final extension at 72 °C for 5 min. Positive (DNA of *A. tholloni* specimens infected by *Ca.* Allocryptoplasma sp.) and negative (water) controls were included in each PCR assay. Following visualization via electrophoresis in 1.5% agarose gel, positive PCR products were sequenced by Eurofins. Sequence chromatograms were cleaned with Chromas Lite (http://www.technelysium.com.au/chromas_lite.html), and alignments were performed using ClustalW, implemented in the MEGA software package (https://www.megasoftware.net/). Alleles of *Ca.* Allocryptoplasma sp. were determined on the basis of sequence identity in nucleotide alignments for 16S rRNA, *sucA*, *groEL*, *rpoB* and *gltA* gene sequences. New sequences obtained in this study were deposited in GenBank with accession numbers OQ724839–OQ724862 and OQ724538–OQ724629.

### Molecular phylogenetic analyses

Phylogenetic analyses were based on sequence alignments of 16S rRNA, *sucA*, *groEL*, *rpoB* and *gltA* gene sequences obtained from analyses of allelic profiles. Sequences of other Anaplasmataceae obtained from GenBank (including representative members of the *Anaplasma*, *Ehrlichia*, *Neorickettsia*, *Wolbachia*, *Neoehrlichia*, *Ca.* Xenohaliotis and *Ca.* Xenolissoclinum) were also included in the phylogenetic analyses. Sequences of other Rickettsiales (including representative members of the families Rickettsiaceae, *Rickettsia rickettsii*, and Midichloriaceae, *Ca.* Midichloria mitochondrii) were used as outgroups. The Basic Local Alignment Search Tool (BLAST; https://blast.ncbi.nlm.nih.gov/blast/Blast.cgi) was used to find additional sequences available on GenBank showing nucleotide similarities with *Ca.* Allocryptoplasma gene sequences. The Gblocks program with default parameters was used to obtain non-ambiguous sequence alignments [6]. All sequence alignments were also checked for putative recombinant regions using the RDP3 analysis package [26]. Tree-based phylogenetic analyses were performed using the maximum-likelihood (ML) method using the MEGA software package (https://www.megasoftware.net/). The evolutionary models that best fit the sequence data were determined using the Akaike information criterion. Clade robustness was assessed by bootstrap analysis using 1,000 replicates.

### Statistical analyses

We tested whether the levels of nucleotide divergence between members of the genus *Ca.* Allocryptoplasma range in threshold values typically observed in other Anaplasmataceae genera. To this end, we computed the nucleotide pairwise identities for each 16S rRNA, *sucA*, *groEL*, *rpoB* and *gltA* sequence dataset (1) between *Ca.* Allocryptoplasma strains found in *I. pacificus*, *I. ricinus*, *A. tholloni*, *A. coelebs* and *H. parmata*, (2) between representative members of the genus *Anaplasma* (GenBank accession numbers: *A. phagocytophilum*, CP006617; *A. platys*, CP046391; *A. capra*, JAOTBF010000000, *A. ovis*, CP015994; *A. marginale*, CP001079; *A. centrale*, CP001759), and (3) between representative members of the genus *Ehrlichia* (*E. chaffeensis*, CP007480; *E. ruminantium*, CP040111; *E. canis*, CP000107; *E. muris*, CP0069017; *E. minasensis*, QOHLL010000000). We further compared intrageneric nucleotide pairwise identities using Wilcoxon signed-rank tests and sequential Bonferroni correction implemented in R (http://www.r-project.org).

## Results

### Diversity of *Ca.* Allocryptoplasma in ticks

We attempted to amplify the 16S rRNA, *sucA*, *groEL*, *rpoB* and *gltA Ca.* Allocryptoplasma gene sequences from the 26 DNA templates belonging to four tick species (Table 1). The five *Ca.* Allocryptoplasma genes were successfully amplified for *A. tholloni* (12 DNA templates) and *I. ricinus* (10), while four *Ca.* Allocryptoplasma genes (16S rRNA, *groEL*, *rpoB* and *gltA*, but not *sucA*) were amplified for *A. coelebs* (1) and *H. parmata* (3) (Table 1). The sequences were easily readable without double peaks, indicating that there was no coinfection of *Ca.* Allocryptoplasma strains in all specimens except two *A. tholloni* specimens. These two specimens showed double peaks for their *sucA*, *groEL* and *rpoB* gene sequences, suggesting that coinfection with at least two distinct genetic variants of *Ca.* Allocryptoplasma may be present in coinfection: double peaks were observed at nucleotide positions that we found variable between the two distinct genetic variants detected in single infection in the 10 other *A. tholloni* specimens (see below). The two *A. tholloni* specimens with possible *Ca.* Allocryptoplasma coinfection were removed from further analyses.

**Table 1:** Allelic profile of the five polymorphic genes for *Ca.* Allocryptoplasma from the four tick species examined in this study (*Ixodes ricinus*, *Amblyomma coelebs*, *A. tholloni*, and *Haemaphysalis parmata*) and from *Ca.* Allocryptoplasma californiense infecting *Ixodes pacificus* available in GenBank. Letters a–g represent the different alleles for each *Ca.* Allocryptoplasma gene. Dashes indicate an absence of gene PCR amplification.


Host species of *Ca.* Allocryptoplasma	*Ca.* Allocryptoplasma genes

16S rRNA	*sucA*	*groEL*	*rpoB*	*gltA*

*Ixodes pacificus* (*Ca.* Allocryptoplasma californiense)	a	a	a	a	a

*Ixodes ricinus*	b	b	b	b	b

*Amblyomma coelebs*	c	c	c	c	_

*Amblyomma tholloni*	d	d, e	d, e	d, e	d

*Haemaphysalis parmata*	e	f	f, g	f, g	_

On the basis of 16S rRNA, *sucA*, *groEL*, *rpoB* and *gltA* gene sequences, we characterized two to six distinct alleles depending on the gene (16S rRNA: 98.7–99.4% pairwise nucleotide identity; *sucA*: 78.3–99.8%, *groEL*: 81.5–99.4%, *rpoB*: 84.0–99.5%, *gltA*: 77.5%), leading to the identification of six genetically different *Ca.* Allocryptoplasma strains in *I. ricinus*, *A. tholloni*, *A. coelebs* and *H. parmata* (Table 1). The highly conserved nature of the 16S rRNA gene prevented molecular distinction of closely related *Ca.* Allocryptoplasma strains, since several *Ca.* Allocryptoplasma strains, identical on the basis of their 16S rRNA gene sequences, could be distinguished through variation in their *sucA*, *groEL* and *rpoB* gene sequences (Table 1). Indeed, two genetically distinct strains of *Ca.* Allocryptoplasma, although identical on the basis of their 16S rRNA gene sequences, were detected in *H. parmata* (Table 1) with high levels of nucleotide identity (16S rRNA: 100% pairwise nucleotide identity; *sucA*: 99.8%, *groEL*: 99.4%, *rpoB*: 98.6%). Two distinct *Ca.* Allocryptoplasma strains were also detected in *A. tholloni* (Table 1; 16S rRNA: 100% pairwise nucleotide identity; *sucA*: 98.1%, *groEL*: 99.1%, *rpoB*: 99.5%, *gltA*: 100%). Of the six *Ca.* Allocryptoplasma strains, each is specific to its respective tick species, and none is shared by two or more tick species (Table 1).

None of the 16S rRNA, *sucA*, *groEL*, *rpoB* and *gltA* gene sequences of *Ca.* Allocryptoplasma strains identified in *I. ricinus*, *A. tholloni*, *A. coelebs* and *H. parmata* were identical to those of *Ca.* Allocryptoplasma californiense infecting *I. pacificus* (Table 1), although showing high levels of nucleotide identity (16S rRNA: 98.8-99.2% pairwise nucleotide identity; *groEL*: 79.4–92.4%, *rpoB*: 84.9–93.6%, *gltA*: 75.1–93.5%). None of the *sucA*, *groEL*, *rpoB* and *gltA* gene sequences observed in this study were 100% identical to other Anaplasmataceae sequences available in GenBank. However, based on partial 16S rRNA gene sequences, the *Ca.* Allocryptoplasma strain found in *I. ricinus* in this study is 100% identical to the *Ca.* Allocryptoplasma strain previously found in *I. ricinus* in France (GenBank accession number: GU734325) [34], Italy (MT829287–MT829288) [27], Serbia (MW900167) [4], Tunisia and Morocco (AY672415–AY672420) [37]. The 16S rRNA gene sequence of the *Ca.* Allocryptoplasma of *I. ricinus* is also 100% identical to infection detected in harvest mites of lizards, *N. autumnalis*, in Italy (MT829286) [27], in striped field mice, *Ap. agrarius* (EF121953–EF121954) [40], and green lizards, *L. viridis* (MG924904) [20], in Slovakia, and in common wall lizards, *P. muralis* (MT829283, MT829285), Italian wall lizards, *P. siculus* (MT829289), and western green lizards, *L. bilineata* (MT829284), in Italy [27]. Furthermore, based on partial 16S rRNA gene sequences, the *Ca.* Allocryptoplasma strains found in *I. ricinus*, *A. tholloni*, *A. coelebs* and *H. parmata* strain are 96.0–99.0% identical to strains found in other ticks, including *H. longicornis* (JN715833, GU075699-GU07504) and *A. dissimile* (MG437272). Further nucleotide BLAST searches found high nucleotide identity (97.5–100%) with the 16S rRNA sequence of an undescribed Anaplasmataceae bacterium (MZ351089) characterized in *A. hebraeum* in Eswatini [24], and with the unpublished sequence of a *Ca.* Allocryptoplasma strain (OM884475) in *I. scapularis* in Florida.

### Phylogeny of *Ca.* Allocryptoplasma

**Figure 1:**
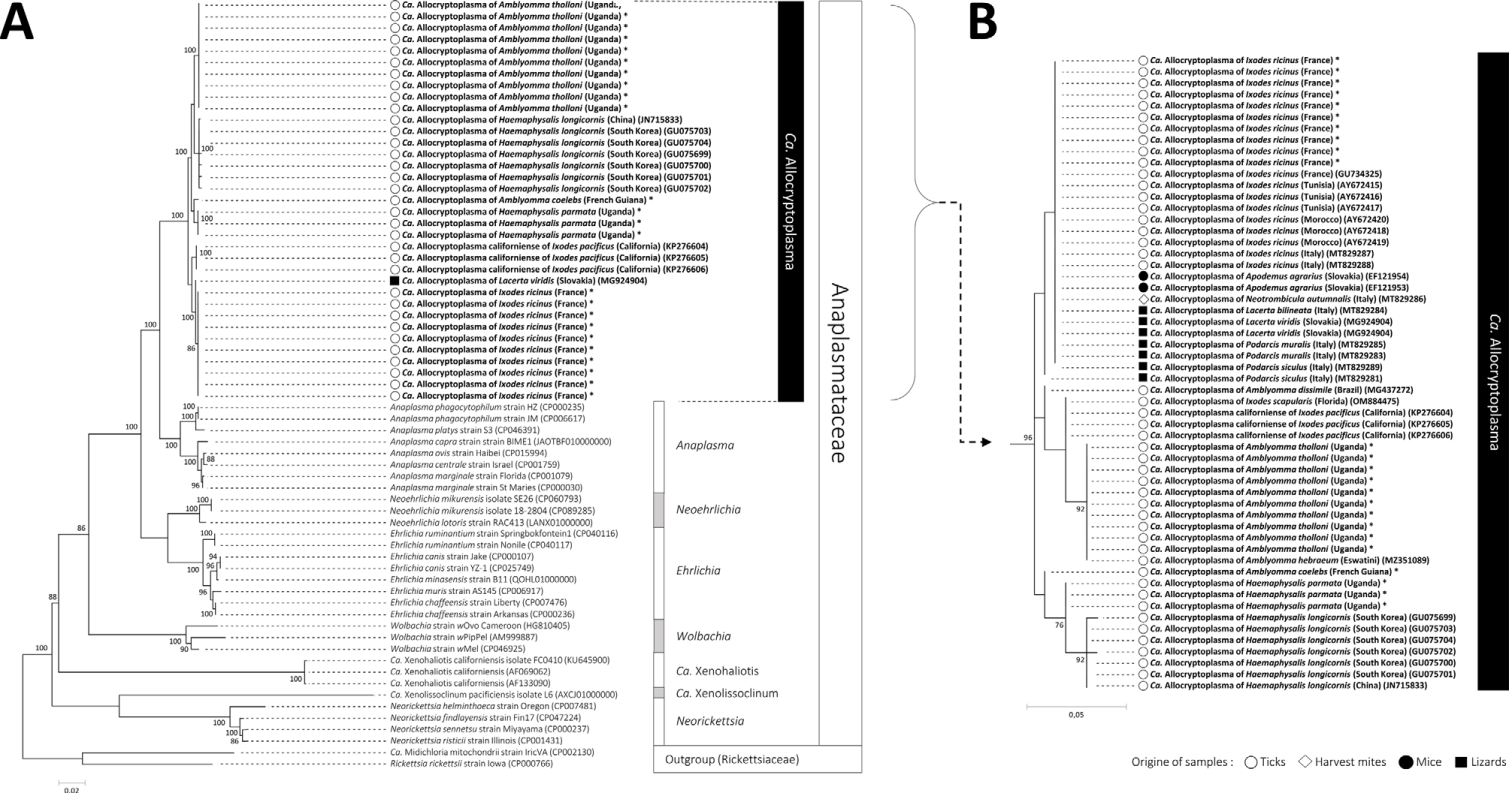
Phylogeny of the family Anaplasmataceae constructed using maximum-likelihood (ML) estimations based on (A) 16S rRNA sequences with a total of 1157 unambiguously aligned bp (best-fit approximation for the evolutionary model: K2+G+I), and (B) on short-length 16S rRNA sequences of *Ca.* Allocryptoplasma with a total of 202 unambiguously aligned bp (best-fit approximation for the evolutionary model: K2+G+I). All genera of the family Anaplasmataceae, including representative species, are indicated. *, *Ca.* Allocryptoplasma sequences obtained in this study (GenBank accession numbers OQ724839–OQ724862). GenBank accession numbers of other sequences used in analyses are shown on the phylogenetic trees. Numbers at nodes indicate percentage support of 1000 bootstrap replicates. Only bootstrap values >70% are shown. The scale bar is in units of substitution/site.

**Figure 2:**
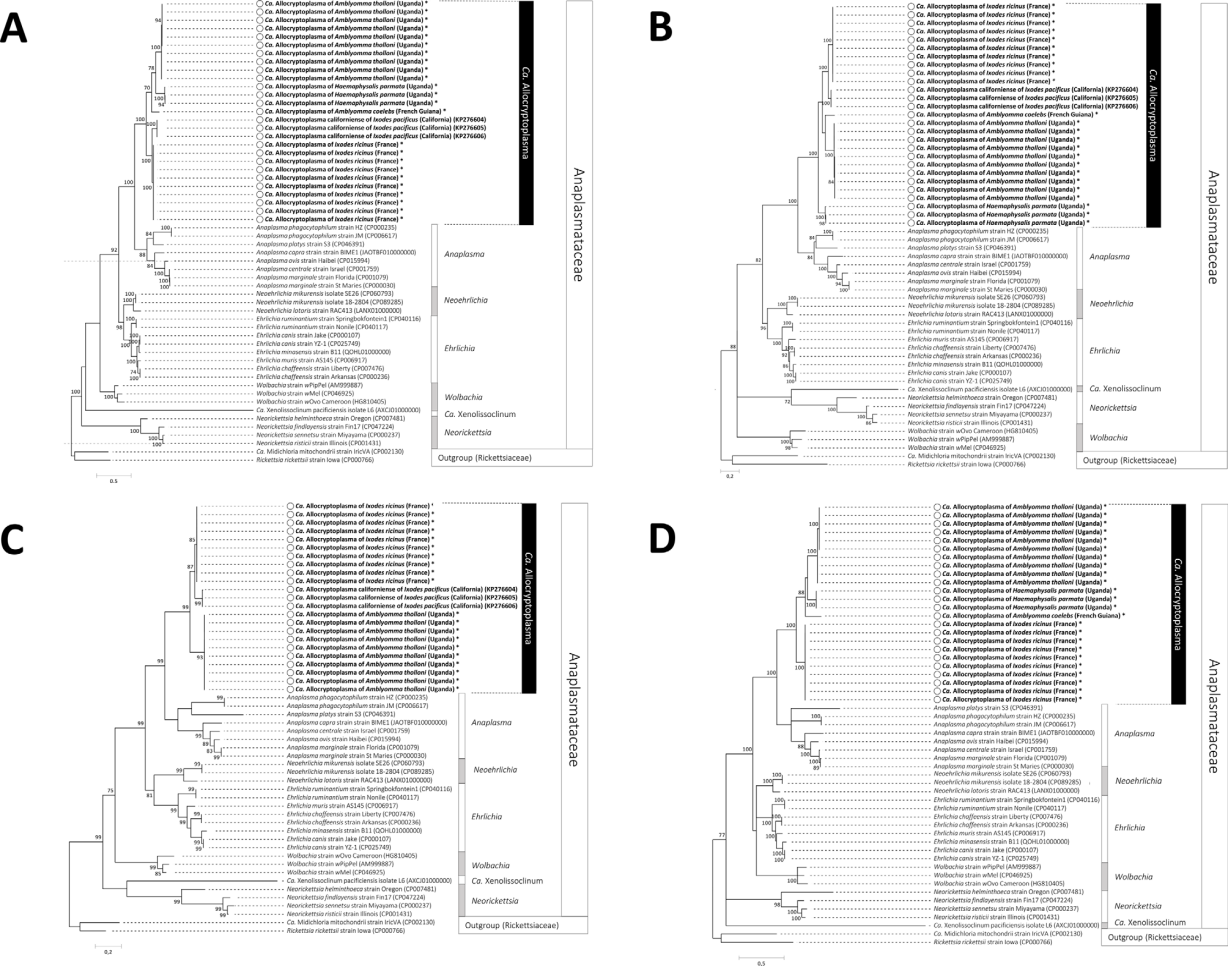
Phylogeny of the family Anaplasmataceae constructed using maximum-likelihood (ML) estimations based on (A) *groEL* gene sequences (529 unambiguously aligned bp; best-fit approximation for the evolutionary model: T92+G), (B) on *rpoB* gene sequences (410 unambiguously aligned bp; best-fit approximation for the evolutionary model: GTR+G+I), (C) on *gltA* gene sequences (289 unambiguously aligned bp; best-fit approximation for the evolutionary model: HKY+G+I), and (D) on *sucA* gene sequences (563 unambiguously aligned bp; best-fit approximation for the evolutionary model: GTR+G+I). All genera of the family Anaplasmataceae, including representative species, are indicated. *, *Ca.* Allocryptoplasma sequences obtained in this study (GenBank accession numbers OQ724538–OQ724629). GenBank accession numbers of other sequences used in analyses are shown on the phylogenetic trees. Numbers at nodes indicate percentage support of 1000 bootstrap replicates. Only bootstrap values >70% are shown. The scale bar is in units of substitution/site.

ML analyses based on 16S rRNA, *sucA*, *groEL*, *rpoB* and *gltA* nucleotide sequences were conducted to examine the phylogenetic proximity between *Ca.* Allocryptoplasma strains and other Anaplasmataceae (Figs. 1 and 2). We observed no sign of recombination in the dataset (all *p* > 0.20 for the GENECONV and RDP recombination detection tests). All phylogenetic reconstructions showed that the *Ca.* Allocryptoplasma strains, including the type species *Ca.* Allocryptoplasma californiense, delineate a robust monophyletic clade within the family Anaplasmataceae (Figs. 1 and 2). The closest relative of the genus *Ca.* Allocryptoplasma is the genus *Anaplasma*: ML analyses based on 16S rRNA, *sucA*, *groEL*, *rpoB* and *gltA* nucleotide sequences consistently showed that *Ca.* Allocryptoplasma and *Anaplasma* are sister genera, while other genera (*Ehrlichia*, *Neoehrlichia*) are more distantly related (Figs. 1 and 2).

Phylogenetic reconstructions showed that the different *Ca.* Allocryptoplasma strains found in the same tick species always cluster together. Indeed, the two *Ca.* Allocryptoplasma strains of *A. tholloni* are more closely related to each other than to any other *Ca.* Allocryptoplasma strain (Figs. 1 and 2). A similar pattern was observed for the two *Ca.* Allocryptoplasma strains found in *H. parmata* (Figs. 1 and 2). The *Ca.* Allocryptoplasma strain of *I. ricinus* is more closely related to *Ca.* Allocryptoplasma californiense of *I. pacificus* and they form together a robust subclade within the genus *Ca.* Allocryptoplasma (Figs. 1 and 2). The *Ca.* Allocryptoplasma strains of *A. tholloni*, *A. coelebs* and *H. parmata* often cluster in ML analyses based on 16S rRNA, *sucA*, *groEL*, *rpoB* and *gltA* nucleotide sequences (Figs. 1 and 2).

Further ML analyses of closely-related 16S rRNA sequences, including sequences from *Ca.* Allocryptoplasma available on GenBank regardless of length (Figs. 1A–1B), confirmed that the *Ca.* Allocryptoplasma strains of other tick species (*I. scapularis*, *H. longicornis*, *A. dissimile*, *A. hebraeum*), harvest mites (*N. autumnalis*), striped field mice (*Ap. agrarius*) and several lizard species (*L. viridis*, *L. bilineata*, *P. muralis* and *P. siculus*) also cluster within the genus *Ca.* Allocryptoplasma. The *Ca.* Allocryptoplasma strain of *I. scapularis* is closely related to *Ca.* Allocryptoplasma californiense of *I. pacificus*, and the *Ca.* Allocryptoplasma strain of *H. longicornis* to the one of *H. parmata* we characterized in this study (Figs. 1A–1B). On the basis of 16S rRNA short-length sequences, the same *Ca.* Allocryptoplasma strain was present in *I. ricinus* from France, Italy, Tunisia and Morocco, as well as in *N. autumnalis* harvest mites from Italy, striped field mice from Slovakia, and lizards from Italy and Slovakia (Fig. 1B).

### Genetic diversity in the genus *Ca.* Allocryptoplasma

**Figure 3:**
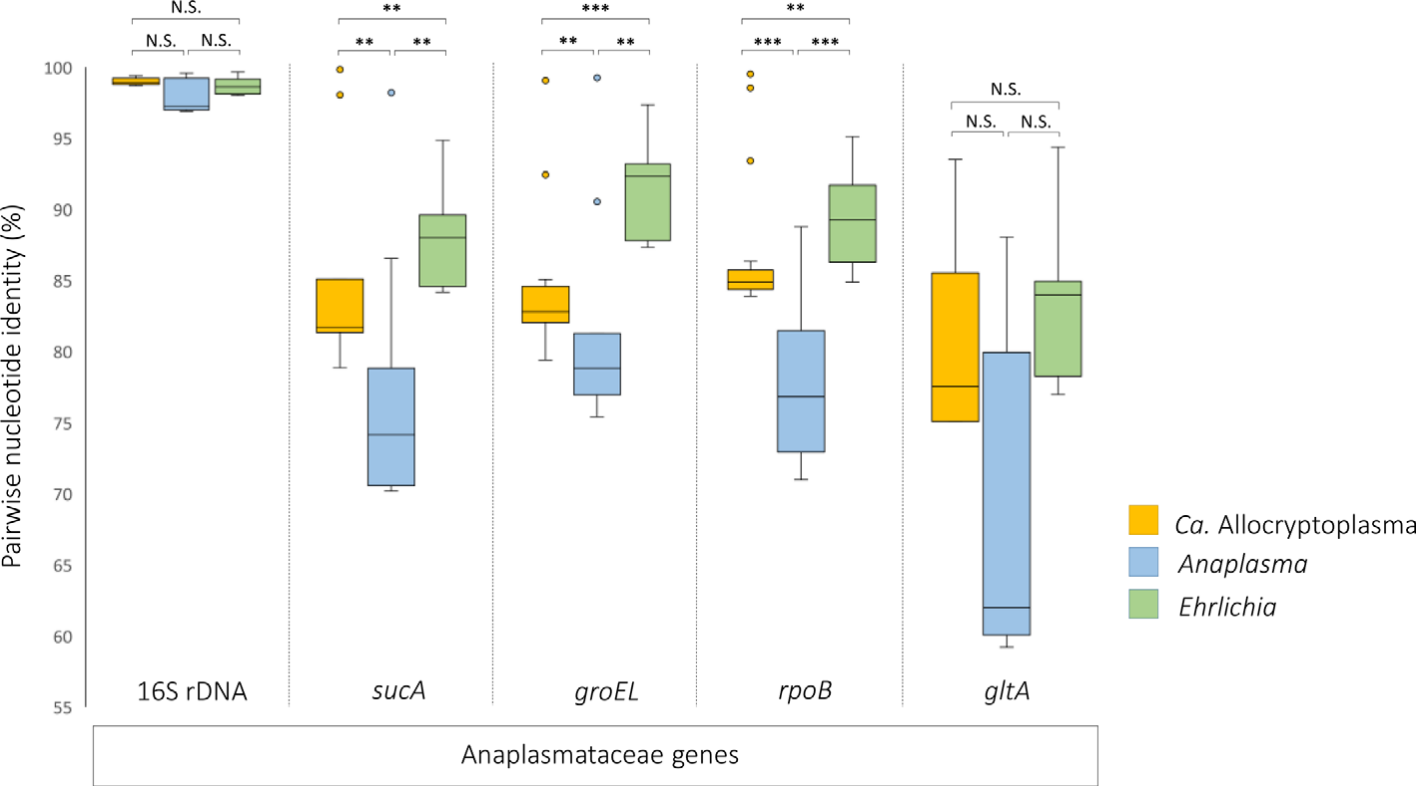
Intrageneric nucleotide pairwise identities of *Ca.* Allocryptoplasma, *Anaplasma* and *Ehrlichia* for the 16S rRNA, *groEL*, *rpoB* and *gltA* gene sequences. Wilcoxon tests: **p < 0.005; ***p < 0.001; N.S., not significant after sequential Bonferroni corrections.

To characterize genetic diversity within the genus *Ca.* Allocryptoplasma, we computed the nucleotide pairwise identities between each *Ca.* Allocryptoplasma strains based on 16S rRNA, *sucA*, *groEL*, *rpoB* and *gltA* nucleotide sequences (Fig. 3). For 16S rRNA and *gltA*, nucleotide pairwise identities within the genus *Ca.* Allocryptoplasma range in threshold values observed within the genera *Anaplasma* and *Ehrlichia* (Wilcoxon tests, all *p* > 0.02, not significant after sequential Bonferroni procedures) (Fig. 3). For *sucA*, *groEL* and *rpoB*, nucleotide pairwise identities within the genus *Ca.* Allocryptoplasma were higher than within the genus *Ehrlichia* (Wilcoxon tests, all *p* < 0.008) but lower than within the genus *Anaplasma* (Wilcoxon tests, all *p* < 0.002) (Fig. 3). This means that *Ca.* Allocryptoplasma has intrageneric genetic diversity similar to values observed in other Anaplasmataceae genera.

## Discussion

In this study, we demonstrated that substantial genetic diversity of *Ca.* Allocryptoplasma is present in ticks in most continents. On the basis of multi-locus gene sequences, we describe six novel distinct genetically different *Ca.* Allocryptoplasma strains in *I. ricinus*, *A. tholloni*, *A. coelebs*, and *H. parmata*. Combining our present study with the current literature sequences indicates that there is molecular evidence of *Ca.* Allocryptoplasma DNA in at least nine tick species in Europe, Asia, Africa, and North and South America, and also in mice and lizards in Europe [4, 14, 20, 22, 27–29, 32, 34, 37, 40]. Phylogenetic and genetic analyses further confirm that these *Ca.* Allocryptoplasma cluster into a monophyletic clade, divergent from all other genera of the family Anaplasmataceae, although more closely related to the genus *Anaplasma*. Their detections in ticks of significant medical or veterinary interest suggest that *Ca.* Allocryptoplasma is an emergent genus of tick-borne pathogens of general concern.

Three complementary lines of argument indicate that *Ca.* Allocryptoplasma is a putative genus similar to validated genera of the family Anaplasmataceae. The first argument lies in the clustering of all *Ca.* Allocryptoplasma strains or species within a unique well-supported clade, a pattern consistently observed in phylogenetic trees based on 16S rRNA, *sucA*, *groEL*, *rpoB* and *gltA* gene sequences. The second concerns the level of genetic diversity in the genus *Ca.* Allocryptoplasma that ranges around values typically observed within the genera *Anaplasma* and *Ehrlichia*. This means that the genetic divergence observed between two members of the genus *Ca.* Allocryptoplasma is on average similar to divergence between two species belonging to another Anaplasmataceae genus. Since only one putative species, *Ca.* Allocryptoplasma californiense [14], has been described within *Ca.* Allocryptoplasma, the observed genetic divergence suggests that additional putative species could be described from genetic data. Finally, analyses of multi-locus gene sequences shows that *Ca.* Allocryptoplasma is a sister genus of *Anaplasma*, i.e., they are more closely related to each other than to any other genus. With the genera *Ehrlichia* and *Neoehrlichia*, *Ca.* Allocryptoplasma and *Anaplasma*, form a monophyletic subgroup of the Anaplasmataceae family specifically associated with ticks and vertebrates.

Tick species often differ in the strains of *Ca.* Allocryptoplasma they harbor. On the basis of multi-locus typing sequences, none of the strains of *Ca.* Allocryptoplasma is shared between different tick species. The best example is found in *H. parmata* and *A. tholloni*, both collected from the same location in Uganda, but each harboring its specific strains of *Ca.* Allocryptoplasma. On the basis of short 16S rRNA sequences, worthy of note is that identical strains of *Ca.* Allocryptoplasma were observed in two cases: one strain in two African tick species, *A. hebraeum* and *A. tholloni*, and another in two North American tick species, *I. pacificus* and *I. scapularis*. However, the 16S rRNA gene sequences show insufficient sequence polymorphism [7], and identical strains of *Ca.* Allocryptoplasma on the basis of their 16S rRNA gene sequences may differ in their sequences for other genes as we observed here in *A. tholloni* and *H. parmata*. Since strains of *Ca.* Allocryptoplasma found in *A. hebraeum* and *I. pacificus* have not been examined for other gene sequences [24], their exact similarity with the strains found in *A. tholloni* and *I. scapularis*, respectively remain uncertain. The multi-locus typing approach can thus reveal greater genetic diversity of *Ca.* Allocryptoplasma than expected by previous studies due to this methodological issue.

The risk of acquiring a *Ca.* Allocryptoplasma infection is currently unknown, but the detection of members of the genus *Ca.* Allocryptoplasma in ticks of significant medical or veterinary interest is of concern. In the Northern Hemisphere, *I. ricinus*, *I. pacificus* and *I. scapularis* are the tick species most commonly biting humans [8, 9], and field specimens of these species harbor *Ca.* Allocryptoplasma [4, 14, 20, 27, 34, 37]. In Western Europe and North Africa, *Ca.* Allocryptoplasma was detected over most of the distribution range of *I. ricinus* [4, 20, 27, 34, 37], suggesting that the infection circulates widely in field populations of this tick species. *Ixodes ricinus*, as *I. pacificus* and *I. scapularis*, are host generalist tick species [8, 9] and they may readily transmit *Ca.* Allocryptoplasma to a range of mammals, birds and reptiles, as suggested by the *Ca.* Allocryptoplasma DNA detected in striped field mice [40] and lizards [20, 27]. In Asia, *H. longicornis* is a livestock pest that transmit pathogens relevant to human and animal health, but it is also an introduced, and now established, exotic species in the western Pacific Region and the USA [43]. In South America, *A. coelebs* is specialized on mammals [5] and can readily bite humans [39], but *A. dissimile* feed exclusively on reptiles and amphibians [5]. In Africa, *H. parmata* is a global generalist, while *A. tholloni* tends to feed on elephants, but both can bite Hominidae, at least occasionally [22]. Humans are therefore exposed to bites of *Ca.* Allocryptoplasma-infected ticks without the risk of infection being documented to date. Currently, there is no specific tests to diagnose *Ca.* Allocryptoplasma infections, suggesting than animal and human cases are undiagnosed, as often shown for novel human pathogens in the family Anaplasmataceae [13, 31, 38]. In this context, new means of detecting and characterizing *Ca.* Allocryptoplasma are now necessary to improve our understanding of *Ca.* Allocryptoplasma epidemiology.

To conclude, we have identified *Ca.* Allocryptoplasma as a clade of genetically diverse, but phylogenetically related bacteria. Our results based on the multi-locus typing scheme suggest that *Ca.* Allocryptoplasma may be a novel valid genus similar to *Anaplasma* and *Ehrlichia* in the family Anaplasmataceae. The repeated detection of *Ca.* Allocryptoplasma in ticks on most continents confirms that infection persists widely through its circulation in at least nine tick species. Additional studies of *Ca.* Allocryptoplasma are needed to determine its transmission cycle and to establish whether these tick-borne bacteria are relevant to human and animal health.

## Supplementary material

The supplementary material of this article is available at https://www.parasite-journal.org/10.1051/parasite/2023014/olm. *Table S1*: List and origin of tick species used in this study for multi-locus typing of *Ca.* Allocryptoplasma.
*Table S2*: Genes and primers used in polymerase chain reaction (PCR) assays for multi-locus typing of *Ca.* Allocryptoplasma.
