# Supplementary material for: Diversity and phylogeny of the tick-borne bacterial genus Candidatus Allocryptoplasma (Anaplasmataceae)
Source: Parasite. 2023 May 10;30:13. doi: 10.1051/parasite/2023014 (PMC10171070; doi:10.1051/parasite/2023014)
Supplement: Supplementary file 1 — Table S1: List and origin of tick species used in this study for multi-locus typing of Ca. Allocryptoplasma. Table S2: Genes and primers used in polymerase chain reaction (PCR) assays for multi-locus typing of Ca. Allocryptoplasma. [file parasite-30-13-s1.pdf]

SUPPLEMENTARY MATERIALS

Diversity and phylogeny of the tick-borne bacterial genus *Candidatus* Allocryptoplasma (Anaplasmataceae)

Table S1. List and origin of tick species used in this study for multi-locus typing of *Ca.* Allocryptoplasma.

Tick species	Origine	n (larvae/nymph /adult)	Reference for tick collection and 16S rDNA metabarcoding
Ixodes ricinus	Niedermunster Forest, France, Europe, 2020	10 (0/0/10)	[3]
Amblyomma coelebs	Montagne de Kaw, French Guiana, South America, 2016	1 (0/1/0)	[1,2]
Amblyomma tholloni	Kibale National Park, Uganda, Africa, 2019-2020	12 (0/12/0)	[4]
Haemaphysalis parmata	Kibale National Park, Uganda, Africa, 2019-2021	3 (0/2/1)	[4]

Table S2. Genes and primers used in polymerase chain reaction (PCR) assays for multi-locus typing of *Ca.* Allocryptoplasma.

Gene	Product	Primers (5'-3')	Fragment size	Reference
16S rrNA	Small ribosomal subunit (SSU rRNA)	Ehr16S_F1 - TCGCTATTAGATGAGCCTA	1st round PCR: Ehr16S_F1 / Ehr16S_R2 : 1200bp	[4]
		Ehr16S_F2 - TGACATGAAGGTCGTATCC	2nd round PCR (fragment 1): Ehr16S_F1 / Ehr16S_R1 : 796bp	
		Ehr16S_R1 - GGTCCAGCCGAACTGACTC	2nd round PCR (fragment 2): Ehr16S_F2 / Ehr16S_R3 : 457bp	
		Ehr16S_R2 - AGCACACCAGCTTCGAGTTA		
		Ehr16S_R3 - AGTTAAGCCAATTCCCATGG		
rpoB	DNA-directed RNA polymerase β chain	Crypto_rpoB_F1 - GATGGRACTCCTGTTGATAT	1st round PCR: Crypto_rpoB_F1 / Crypto_rpoB_R2 : 536bp	This study
		Crypto_rpoB_F2 - CTGTTGATATTRTRCTTAATTC	2d round PCR: Crypto_rpoB_F2 / Crypto_rpoB_R1 : 469bp	
		Crypto_rpoB_R1 - GGACCNACAGAYCTWGCATGTA		
		Crypto_rpoB_R2 - CGYTGMCCACCAAAATGAGA		
sucA	Alpha-ketoglutarate dehydrogenase	Crypto_sucA_F1 - GTTATGGGNTTTGAGTAYGG	1st round PCR: Crypto_sucA_F1 / Crypto_sucA_R2 : 636bp	This study
		Crypto_sucA_F2 - TGGWGATTTTGCWAATGGTGC	2d round PCR: Crypto_sucA_F1 / Crypto_sucA_R1 : 616bp	
		Crypto_sucA_R1 - CTACCTCTGCATTCTGATA	Alternative 2d round PCR: Crypto_sucA_F2 / Crypto_sucA_R1 : 548bp	
		Crypto_sucA_R2 - GGGCTCTTCYTGRCACCA		
groEL	Chaperone protein GroEL (Hsp60)	Crypto_GroEL_F1 - CCTTCYTCAACAGCAGCYCTAG	1st round PCR: Crypto_GroEL_F1 / Crypto_GroEL_R2 : 713bp	This study
		Crypto_GroEL_F2 - ACAGCAACVCCACCWGAWAGCTT	2d round PCR: Crypto_GroEL_F2 / Crypto_GroEL_R1 : 572bp	
		Crypto_GroEL_R1 - ACYGATGGTATGCAGTTTGA		
		Crypto_GroEL_R2 - ACNGTTGAAGARAGTAARGG		
gltA	Citrate synthase	Crypto_gltA_F1 - ATTACGTTTATWGAYGGGGAT	1st round PCR: Crypto_gltA_F1 / Crypto_gltA_R2 : 1011bp	This study
		Crypto_gltA_F2 - GTTATAGAGGATATGACATAG	2d round PCR: Crypto_gltA_F2 / Crypto_gltA_R2 : 977bp	
		Crypto_gltA_F3 - AGAACTTTNTTCACATGATGTT	Alternative 2d round PCR: Crypto_gltA_F3 / Crypto_gltA_R2 : 606 bp	
		Crypto_gltA_R2 - AATYTCATACCATTGAGCAGAC		

References

1. Binetruy F, Buysse M, Lejarre Q, Barosi R, Villa M, Rahola N, Paupy C, Ayala D, Duron O. 2020. Microbial community structure reveals instability of nutritional symbiosis during the evolutionary radiation of Amblyomma ticks. Molecular Ecology, 29, 1016–1029.
2. Binetruy F, Chevillon C, Thoisy B de, Garnier S, Duron O. 2019. Survey of ticks in French Guiana. Ticks and Tick-Borne Diseases, 10, 77–85.
3. Boyer PH, Barthel C, Mohseni-Zadeh M, Talagrand-Reboul E, Frickert M, Jaulhac B, Boulanger N. 2022. Impact of Different Anthropogenic Environments on Ticks and Tick-Associated Pathogens in Alsace, a French Region Highly Endemic for Tick-Borne Diseases. Microorganisms, 10, 245.
4. Lacroux C, Bonnet S, Pouydebat E, Buysse M, Rahola N, Rakotobe S, Okimat J-P, Koual R, Asalu E, Krief S, Duron O. 2023. Survey of ticks and tick-borne pathogens in wild chimpanzee habitat in Western Uganda. Parasites & Vectors, 16, 22.
